# Fertilization impacts microbiomes along the grassland trophic chain

**DOI:** 10.1093/ismeco/ycaf162

**Published:** 2025-09-16

**Authors:** Karoline Jetter, Kunal Jani, Kerstin Wilhelm, Ulrike Stehle, Rostand R Chamedjeu, Christian U Riedel, Lena Wilfert, Patrick Schäfer, Simone Sommer

**Affiliations:** Microbial Biotechnology, Department of Biology, University of Ulm, Albert-Einstein-Allee 11, 89081, Ulm, Germany; Institute of Evolutionary Ecology and Conservation Genomics, University of Ulm, Albert-Einstein Allee 11, 89081, Ulm, Germany; Institute of Evolutionary Ecology and Conservation Genomics, University of Ulm, Albert-Einstein Allee 11, 89081, Ulm, Germany; Institute of Evolutionary Ecology and Conservation Genomics, University of Ulm, Albert-Einstein Allee 11, 89081, Ulm, Germany; Institute of Evolutionary Ecology and Conservation Genomics, University of Ulm, Albert-Einstein Allee 11, 89081, Ulm, Germany; Microbial Biotechnology, Department of Biology, University of Ulm, Albert-Einstein-Allee 11, 89081, Ulm, Germany; Institute of Evolutionary Ecology and Conservation Genomics, University of Ulm, Albert-Einstein Allee 11, 89081, Ulm, Germany; Microbial Biotechnology, Department of Biology, University of Ulm, Albert-Einstein-Allee 11, 89081, Ulm, Germany; Institute of Evolutionary Ecology and Conservation Genomics, University of Ulm, Albert-Einstein Allee 11, 89081, Ulm, Germany; Institute of Evolutionary Ecology and Conservation Genomics, University of Ulm, Albert-Einstein Allee 11, 89081, Ulm, Germany; Institute of Zoology, Regensburg University, Universitätsstraße 31, 93053, Regensburg, Germany; Institute of Phytopathology, iFZ Research Centre for Biosystems, Land Use and Nutrition, Justus Liebig University, Heinrich-Buff-Ring 26, 35392, Gießen, Germany; Institute of Evolutionary Ecology and Conservation Genomics, University of Ulm, Albert-Einstein Allee 11, 89081, Ulm, Germany

**Keywords:** fertilization, organic fertilizer, microbiome, 16S rRNA, grassland, land use, trophic chain, trophic interaction, one health, ecosystem monitoring

## Abstract

Agricultural grasslands are often managed intensively, influencing soil properties and microbial communities. These changes may, in turn, affect the microbiome of organisms across multiple trophic levels within the same habitat, and significant shifts in these communities can disrupt health and functionality along the entire trophic chain. This study investigates how fertilization affects microbial communities in multiple connected below- and above-ground trophic compartments of grassland ecosystems. We compared control grassland sites to those treated with organic fertilizers—biogas digestate, cow/horse manure, and pig slurry—using 16S rRNA amplicon sequencing and soil nutrient analysis. Shifts in microbial composition occurred in response to fertilization, with compartment-dependent effects. Changes were more pronounced in belowground compartments, with pig slurry fertilization exhibiting the most substantial impact. Overlapping bacterial genera detected among soil, roots, and higher trophic levels show the potential strong interactions across trophic levels shaping microbial communities. Pig slurry-derived microbial taxa were found in all compartments, but their low prevalence suggests an indirect effect of fertilization, primarily due to changes in nutrient availability. Compared to the control sites, pig slurry-fertilized sites showed proliferation of certain taxa, including *Clostridium, Ruminococcus* or *Lachnoclostridium*, particularly in the animal compartments. Our study highlights that the effects of fertilization permeate all trophic levels, with potential ecological and health implications aligned with the One Health framework.

## Introduction

On a global scale, intensive land use is a major driver of biodiversity loss and ecosystem dysfunction [1, 2]. Apart from croplands, grasslands are the main component of agricultural landscapes in temperate regions, providing forage for grazers such as cattle and sheep. To increase the yield of hay and silage for livestock feeding, livestock slurry or manure are widely used fertilizers in grassland ecosystems. These organic fertilizers improve soil nutrient content and resource availability, thereby increasing plant productivity [3]. However, organic fertilizers not only affect soil properties directly through nutrients or pH changes, but also add livestock-associated microbial communities [4, 5]. This microbial influx may displace natural members of the microbiota and introduce additional microbes with undesirable traits e.g. antibiotic resistance genes (ARGs) [6–8]. As microbial communities are known to overlap between organisms within the same habitat [9], such effects may extend to different trophic levels for e.g. from soil to higher trophic compartments—impacting both microbiomes and macrofauna, along with associated ecosystem functions. However, the extent of these impacts along the trophic chain remains largely undocumented [10, 11].

Biogas digestate, cattle manure, and pig slurry are common organic fertilizers that vary both in their ability to promote plant growth and in their ecological impacts. Biogas digestate has a low carbon-to-nitrogen ratio that supports microbial and plant productivity without depleting soil organic carbon, contributing to agroecological sustainability [12]. However, it can retain contaminants from feedstock, including antibiotics, heavy metals, and pathogens [13]. Cattle manure, rich in organic carbon and nutrients, introduces intestinal microbiota that often fail to persist in soil but may include pathogens like *E. coli* O157: H7 and *Salmonella*, which can survive long after application [14]. Pig slurry, a nitrogen-rich fertilizer, is linked to high levels of ARGs and pathogens, largely due to intensive pig farming practices where animals are housed in confined conditions that increase infection risk [15, 16]. Although pre-treatments such as composting, anaerobic digestion, and on-farm storage are commonly applied, the initial microbial composition of pig slurry remains a critical factor influencing its ecological impact [16]. On-farm storage may facilitate the survival and adaptation of gut-associated microbes, enabling their persistence in external environments, particularly in grassland soils. Unlike manure from grazing cattle or horses, which is deposited directly onto pastures, pig slurry thus may introduce a higher proportion of allochthonous microbes. Furthermore, the routine therapeutic use of antibiotics in intensive pig farming may contribute to the influx of antibiotic residues, abundant ARGs, and potential pathogens in the environment. Collectively, these factors suggest that pig slurry may exert the most pronounced influence on soil microbial communities and ecosystem processes.

Given the substantial microbial inputs and varying ecological footprints of these organic fertilizers, their application can significantly influence soil microbial communities and downstream ecosystem processes. There is growing evidence that intensification of land use and fertilization affects bacterial and fungal communities in soil, roots, as well as flowers and leaves, reducing the complexity of microbiome networks and causing microbial communities to become more homogeneous [17–20]. The diversity and stability of host-associated microbial communities, such as root-associated microbiomes in plants and gut microbiomes in animals, are crucial for host health [21–23]. These communities contribute to essential ecosystem services [7, 21, 24, 25] or act as a barrier to the accumulation of antimicrobial resistances in the environment [26]. Disturbance of the microbiome might shift microbial composition and diversity, leading to dysbiosis characterized by a loss of commensals and a rise in pathogens, with potentially serious consequences for ecosystem function and host health. Therefore, based on the eco-holobiont framework and the One Health concept, it is important to move beyond examining specific compartments in isolation and instead consider how environmental perturbations like fertilization affect interconnected biotic and abiotic components across the trophic chain [7, 11, 27, 28].

Although aboveground and belowground communities may be differentially affected by land use changes, they are ecologically interlinked through plant-mediated resource flows that define typical grassland trophic chains [29]. Soil microbes play a central role in this system by decomposing organic matter, recycling nutrients, promoting plant growth, and enhancing stress resilience. These interactions make soil–plant feedbacks essential for the functioning and stability of grassland ecosystems [30, 31]. In turn, plants shape microbial communities by supplying resources and selectively recruiting beneficial microbes through rhizodeposition into the rhizosphere [23, 32, 33]. Various detritivorous and herbivorous animal species feed on belowground soil organic matter, roots and plant litter, as well as aboveground plant biomass, thus ingesting soil- and plant-associated microbes into their gut [34, 35]. Similar to soil and rhizosphere, these (macro)organisms are also affected by fertilization-driven disturbances and may serve as reservoirs and vectors for zoonotic pathogens and antibiotic resistant bacteria [8, 25, 36]. Flowers can act as reservoirs for microbes [37]. As different pollinating insects share floral resources, flowers can function as transmission sites for microbes in pollinators, making microbe transfer between flowers and pollinators bidirectional [38, 39]. These trophic interactions, and the importance of stable and balanced microbial communities for host and environmental health, reinforce the need to collectively study microbial communities associated with multiple links in the trophic chain to understand the impact of fertilizers on environmental health and ecological function. However, most studies investigating the effects of land use and fertilizers on microbial species richness and diversity have focused on individual trophic levels in isolation, rather than considering the broader community of interacting above- and belowground trophic levels [1, 40, 41].

In this study, we investigated the effects of fertilization on microbial diversity, composition, and interconnectedness along the trophic chain—a linear network of interactions in a food web, beginning with producers and extending to consumers—within grassland ecosystems. In summer 2022, 494 samples were collected from seven trophic compartments across agricultural grasslands on the Swabian Alb (Germany), subjected to four fertilization regimes: control, cow and horse manure, biogas digestate, and pig slurry. Samples were categorized into the plant-related “phytosphere” and animal-related “zoosphere,” with further distinction between aboveground and belowground compartments. Specifically, we aimed to investigate: (i) whether fertilization exerts similar effects on microbiomes across above- and belowground, as well as plant- and animal-associated compartments of the trophic chain, and (ii) whether fertilization-driven microbial modulation shapes microbiomes across interconnected trophic compartments. We hypothesized that belowground communities with higher exposure to fertilization would be most affected, with pig slurry inducing the strongest impact by not only enriching soil nutrient reserves but also introducing elevated levels of ARGs and potential pathogens.

Our findings show that fertilization significantly affects belowground microbial diversity and composition across all trophic compartments, with pig slurry exerting the most pronounced influence. The limited abundance of pig slurry-derived taxa across trophic compartments, coupled with the substantial effect of soil nutrient content, suggests that these changes were primarily driven by altered soil nutrient levels rather than the establishment of slurry-derived taxa. The host-dependent nature of these responses highlights the importance of assessing fertilization impacts across trophic compartments within a One Health framework.

## Materials and methods

### Study area and sample collection

Sample collection was carried out between May and July 2022 on agricultural grassland sites located on the Swabian Alb, Germany (see Fig. S1A) that were subjected to four different fertilization regimes typical for this region i.e. fertilization with (i) biogas digestate, (ii) cow/horse manure, (iii) pig slurry, and (iv) control sites, i.e. former military sites that are minimally managed by grazing flocks of sheep (maximum twice per year). In total, 28 grassland sites were investigated, including seven control, eight biogas digestate, seven cow/horse manure, and six pig slurry fertilized sites. Sampling specimens were selected by targeting organisms that are representative of different trophic compartments above and belowground (Fig. S1B) containing animals (zoosphere; i.e. feces of earthworms and voles (*Microtus arvalis, Arvicola amphibious*), bumblebee gut contents [*Bombus lapidarius*]) and plant-related samples (phytosphere; i.e. endosphere, rhizosphere and flowers of *Trifolium pratense*), as well as soil and samples from pig slurry. *T. pratense* L. is an important legume forage crop in temperate regions due to its high protein content [42] and the symbiosis with nitrogen fixing bacteria [43]. As *T. pratense* flowers are frequently visited by bumblebees and honeybees [44] and as several vole species prefer *T. pratense* roots as a food source [45–47], this system links above and belowground compartments in a widely distributed trophic chain in grassland ecosystems. In addition to microbial analyses, soil nutrient content and plant growth parameters (aboveground and root biomass, flower number, and plant height) were measured. Nitrogen input was estimated using the Biodiversity Exploratories land use index [48]. Detailed sampling and processing methods are provided in the supplement.

### DNA extraction, library preparation, and 16S rRNA gene amplicon sequencing

A total of 494 samples were collected (see Table S1 for sample sizes per compartment). To assess the bacterial composition, the V4 hypervariable region of the 16S rRNA gene was amplified in a two-step polymerase-chain reaction (PCR) using the primers 515 F (5′-GTGCCAGCMGCCGCGGTAA-3′) and 806 R (5′-GGACTACHVGGGTWTCTAAT-3′) [49]. To avoid amplifying high amounts of chloroplast and mitochondrial DNA, peptide nucleic acid (PNA)-DNA clamps (mPNA and pPNA for plant [endosphere, flower] and mPNAs [bumblebee] samples) were added to the first PCR reaction [50]. Paired-end sequencing of the constructed libraries was conducted in two separate runs on an in-house Illumina MiSeq sequencing platform according to Fackelmann et al. [51] and Heni et al. [52] (see supplement for details). DNA extraction and sequencing of a total of 494 samples (excluding negative and positive control) resulted in 10 044 014 reads and 27 753 ASVs after filtering.

### Soil nutrient assessment by electro-ultrafiltration

Soil samples were pooled per sampling site (n = 23) for nutrient content analysis by the Bodengesundheitsdienst GmbH (https://www.bodengesundheitsdienst.de). Using electro-ultrafiltration (EUF), the concentrations of macronutrients (nitrate, organic nitrogen, phosphorus, potassium, sulfur, boron, magnesium, and calcium), micronutrients (iron, manganese, zinc, and copper), and sodium were determined for each site.

### Bioinformatic sequence processing and statistical analyses

Sequence processing—removal of primers, denoising, removal of chimeras and merging of paired-end reads [53]—was conducted using the DADA2 pipeline in QIIME2 (version 2022.8) [54], (see supplementary material). All statistical analyses were performed in R and R Studio [55]. To evaluate the impact of fertilization on the microbiomes of all seven different trophic compartments, measures for microbial alpha diversity (Shannon index) and beta diversity (Bray-Curtis distances) were calculated for each compartment separately. The differences between the fertilization regimes within each compartment were evaluated using ANOVA with Tukey’s HSD post-hoc test for alpha diversity and pairwise PERMANOVA (999 permutations) for beta diversity. The latter was conducted using the vegan package [56] and the pairwise Adonis package [57].

To determine how soil nutrients influence microbial composition, redundancy analysis (RDA) was performed using Bray–Curtis distances and available nutrients (P, K, S, N, NO₃, Na, Ca, Mg, B, Mn, Zn, Cu, Fe) as constraints. The ordiR2step function (vegan package) was used for forward selection to identify nutrients significantly explaining microbial variation. Using the ordinate function (microviz package) [58], we ran separate RDA models for macronutrients, micronutrients, and all nutrients to evaluate their impact on microbial community differences. Differences in nitrogen input and plant growth parameters across field sites were assessed using Kruskal–Wallis tests followed by pairwise Wilcoxon tests.

Differentially abundant taxa, i.e. taxa that respond to fertilization within the different trophic compartments, were identified using Analysis of Composition of Microbiomes (ANCOMBC2) [59, 60]. First, samples from all three fertilization regimes were compared individually against those from the control sites. Samples from all fertilization regimes were then compared against each other (all pairwise comparisons). Core microbiome analyses were conducted for each trophic compartment individually, encompassing all pairwise comparisons between fertilization regimes to determine resilient taxa, i.e. taxa present in all samples within a compartment regardless of the fertilization regime, utilizing the core function in the microbiome package (detection cut-off: 0.0001, prevalence cut-off: 0.5) [61].

Subsequent analyses were conducted on a refined dataset, comprising only those ASVs identified as responding taxa in the differential abundance analysis and as resilient taxa in the core microbiome analysis. To verify the impacts of fertilizer application on microbial composition within the various trophic compartments, the refined dataset was employed to conduct a canonical analysis of principal coordinates (CAP) [62], followed by PERMANOVA and pairwise PERMANOVA. Based on interactions among trophic compartments, a Source Tracker2 [63] analysis was conducted to assess microbial taxa that overlap between compartments and to elucidate potential exchanges of taxa between compartments. All graphs were created in R using ggplot2 [64].

## Results

### Impact of fertilization on microbial alpha and beta diversity in different compartments along the trophic chain

**Figure 1:**
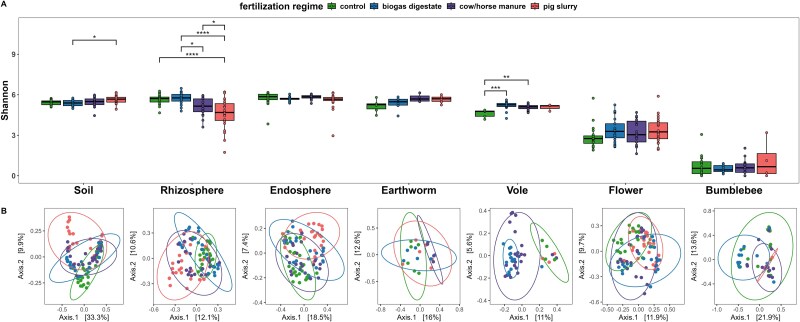
Effects of fertilization on microbial diversity. (A) Alpha diversity (Shannon index) and (B) Beta diversity (Bray-Curtis) measures for individual compartments along the trophic chain. ANOVA followed by post hoc Tukey’s HSD test was used to analyze effects of fertilization regimes on alpha diversity (^*^ = *P = .*05, ^**^P < .01, ^***^P < .001 ^****^P < .0001). Differences in beta diversity were assessed using PERMANOVA followed by pairwise PERMANOVA.

Microbial alpha (ANOVA*:* Shannon*, P < .*001) and beta diversity (PERMANOVA: Bray–Curtis, df = 3; R^2^ = 0.027, *P = .*001) were highly variable between trophic compartments and fertilization regimes (Fig. 1). Disentangling the overall significant effect of fertilizer application on alpha diversity revealed that Shannon diversity was significantly impacted by fertilization in the belowground compartments—soil (ANOVA: *P = .*029) and rhizosphere (ANOVA: *P < .*001), as well as in the belowground zoosphere compartment voles (ANOVA: *P = .*001) (Fig. 1A, Table S2). In soil samples, alpha diversity was increased in pig slurry fertilized sites when compared to biogas but not to control or cow/horse manure fertilized sites (TukeyHSD: biogas vs. pig: *P = .*029) (Table S2). For voles, alpha diversity was increased in biogas- and cow/horse-manure-fertilized sites (TukeyHSD: control vs. biogas: *P < .*001; control vs. cow/horse manure: *P = .*003) (Table S2). Moreover, alpha diversity of the rhizosphere was significantly lower in pig slurry-fertilized sites as compared to all other treatment groups (TukeyHSD: control vs. pig: *P < .*001; biogas vs. pig: *P < .*001, cow/horse manure vs. pig*: P = .*049; biogas vs. cow/horse manure: *P = .*026) (Table S2). Flowers and bumblebees, representing the aboveground trophic compartments, as well as earthworms exhibited no differences in alpha diversity (Fig. 1A). Similarly, the root endosphere showed no significant variation under the different fertilization regimes (Table S2). Hence, the impact of fertilizer application is more pronounced in belowground compartments.

Beta diversity was significantly affected by fertilization regimes in all trophic compartments (Fig. 1B). Within the phytosphere and soil compartments, we noted significant differences in beta diversity revealing an impact of all four fertilization regimes on microbiome compositions (soil: *P = .*001, rhizosphere: *P = .*001, endosphere: *P = .*001, flower: *P = .*001; PERMANOVA: Bray-Curtis) (Table S3). Similarly, differences in beta diversity between fertilization regimes were observed in all zoosphere compartments (voles: *P = .*001; earthworms: *P = .*005; bumblebees: *P = .*001 PERMANOVA: Bray-Curtis) (Table S3). In voles, microbial composition differed between all fertilization regimes except for comparisons between control and pig slurry fertilized sites (pairwise PERMANOVA: *P = .*656) (Table S3). Pig slurry and cow/horse manure fertilization altered earthworm microbiomes (pairwise PERMANOVA: control vs. pig: *P = .*021; control vs. cow: *P = .*002) (Table S3) and fertilization with biogas digestate impacted the microbial compositions in bumblebees (pairwise PERMANOVA: control vs. biogas: *P = .*023; cow vs. biogas: *P = .*001; pig vs. biogas: *P = .*005) (Table S3). Overall, these analyses revealed an impact of fertilization on microbial communities of all trophic compartments and pig slurry appeared to have the most pronounced effects.

### Effects of soil nutrients on soil microbial communities and plant growth

**Figure 2:**
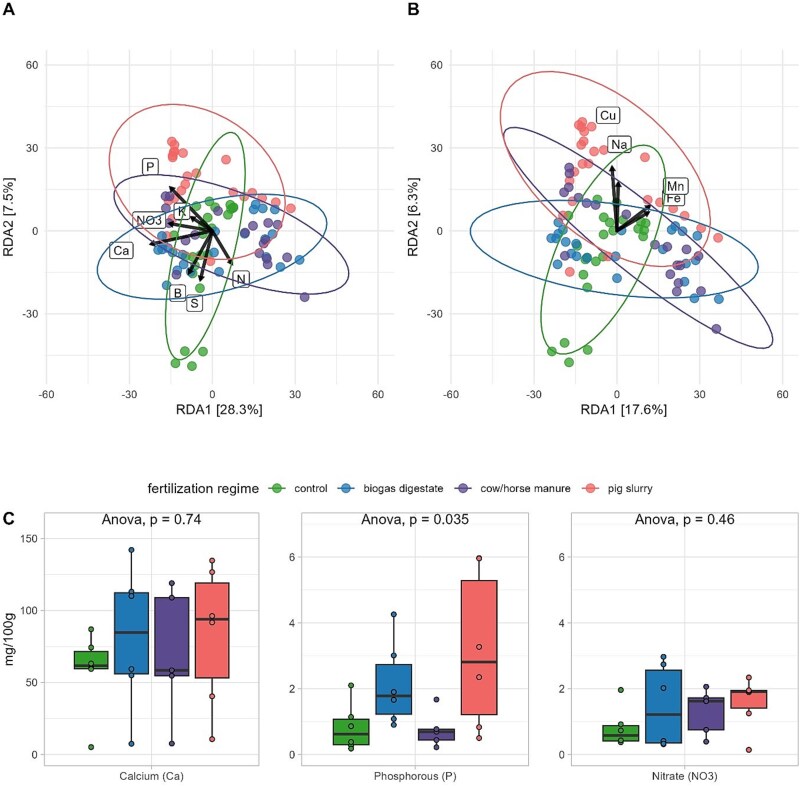
Soil nutrient content analysis. RDA showing the effects of (A) macronutrients and (B) micronutrients on Bray–Curtis distances of soil microbial communities. Arrows indicate the direction and identity of soil nutrients associated with microbial community variation. Colors represent fertilization regimes. (C) Soil concentrations of the three key nutrients (Ca, P, and NO₃^−^) driving microbial community differences are shown, with ANOVA assessing differences between fertilization treatments.

RDA was used to model the amount of variation between soil microbial communities of different samples based on Bray–Curtis distances that can be explained by the different soil nutrients. Soil electro-ultrafiltration provided the content of eight macronutrients (P, K, S, N, nitrate, Ca, Mg, B) and four micronutrients (Mn, Zn, Cu, Fe) as well as the sodium content in soil samples. Forward selection of significant values revealed non-significant impacts of zinc and magnesium. After selecting significantly impacting nutrients, the model explained 51.9% of variation between the different soil microbial communities. Selected macronutrients explained 38.9% of variation (adjusted R^2^ = 0.389) (Fig. 2A) while selected micronutrients including sodium explained 28.2% of variation (adjusted R^2^ = 0.282) (Fig. 2B) between microbial communities of different samples. Out of all tested nutrients, calcium explained the most variation (19.2%), followed by phosphorous (11.1%) and nitrate (9.1%), while only phosphorous contents were significantly different between soils collected on the four different fertilization regimes (ANOVA: *P = .*035) (Fig. 2C).

Nitrogen input (kg[N]) per field site was calculated based on landowner information (Fig. S2A). Inputs varied significantly across fertilization regimes (Kruskal-Wallis, *P < .*001), with all pairwise comparisons significant: control sites had the lowest input, followed by cow/horse manure, while biogas and pig slurry sites received the highest nitrogen levels (Table S4). To assess the impact on plant growth, we measured aboveground dry biomass, root fresh biomass, plant height, and flower number in *T. pratense* (Fig. S2B). Aboveground biomass (*P = .*200) and flower number (*P = .*610) did not differ significantly between treatments. However, root biomass differed between control and cow/horse manure sites (*P = .*046), and plant height showed overall differences (*P < .*001), being significantly greater on biogas and pig slurry sites compared to control and cow/horse manure sites.

### Fertilization impacts on resilient and responding bacterial taxa along trophic compartments

**Figure 3:**
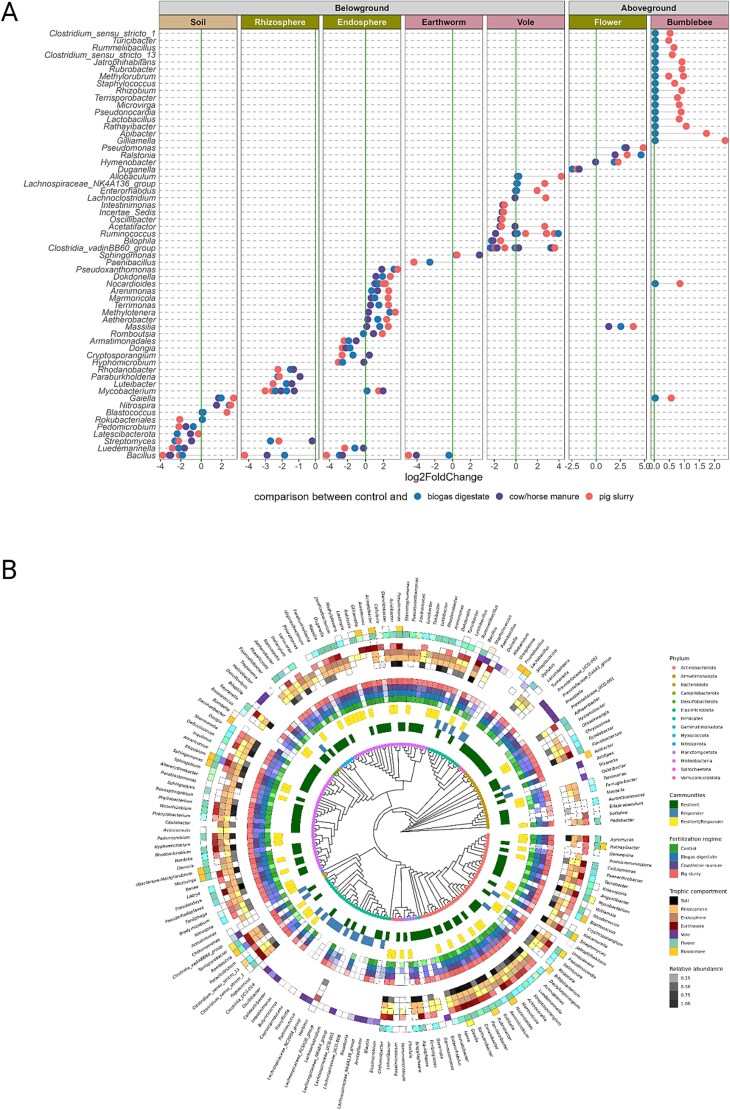
Bacterial genera and taxa responding to fertilization within the different trophic compartments. (A) Differentially abundant genera between unfertilized control sites (vertical line) and sites fertilized with biogas digestate, cow/horse manure and pig slurry, respectively, were identified using ANCOMBC2. Phytosphere, zoosphere compartments, and soil are marked with different colors in the title bar. Positive values of log2fold change represent genera that are more abundant on fertilized sites; negative values represent genera that are less abundant on fertilized sites compared to control sites. (B) Responding (differentially abundant) and resilient taxa (members of the core microbiome) according to fertilization regimes and trophic compartments they were found in.

We assessed the impact of fertilization on microbial communities across trophic compartments using ANCOM-BC2, identifying 600 differentially abundant ASVs from 82 genera. These were classified as *responder taxa*—taxa whose abundances significantly changed in response to fertilization, indicating sensitivity to nutrient input (Fig. 3A, Fig. S3). To identify taxa persisting across treatments, we performed a core microbiome analysis (prevalence ≥0.5, detection ≥0.0001) within each compartment, identifying 894 ASVs from 159 genera classified as *resilient taxa* (Fig. S4). Comparison revealed 57 overlapping genera, termed *fertilization-responsive core genera*—taxa consistently present yet responsive to fertilization, suggesting ecological adaptability (Fig. 3B).

In the phytosphere (excluding flowers), soil, and earthworms, *resilient* genera were more abundant than *responders*, and all detected *responder* genera were subsets of the *resilient* group (Table S5). This suggests that fertilizer-induced compositional changes in these compartments were primarily driven by changes in the abundance of *resilient-taxa*. In contrast, bumblebees and voles harbored fewer *resilient* than *responder* genera, with limited overlap between the two groups, indicating that microbial shifts in these compartments were likely driven by opportunistic, non-core taxa (Table S5).

Seven *responder* genera were shared across at least two trophic compartments (Fig. 3A). Among these, *Bacillus, Luedemannella,* and *Streptomyces* consistently decreased in abundance at fertilized sites in soil, roots, and earthworms. In contrast, *Gaiella, Massilia,* and *Nocardioides* were enriched at fertilized sites in soil, endosphere, flowers, and bumblebees (Fig. 3A). Among *resilient-taxa*, five genera were core to multiple compartments. Notably, *Bacillus, Microvirga, Pseudonocardia,* and *Gaiella* were prevalent in all belowground and plant-associated compartments (soil, rhizosphere, endosphere, earthworm gut, and flower) (Fig. S4). *Sphingomonas* was also core to the rhizosphere, endosphere, earthworm gut, flower, and bumblebee microbiomes. Voles exhibited no overlapping core genera, indicating a highly host-specific microbiome (Fig. S4).

Importantly, *Bacillus* and *Gaiella* emerged as key fertilization-responsive core genera- both core to multiple compartments and differentially abundant. *Bacillus* declined with fertilization, suggesting that it is negatively affected by nutrient enrichment, while *Gaiella* increased, indicating a potential advantage under fertilized conditions.

### Identification of overlapping taxa between different trophic compartments

**Figure 4:**
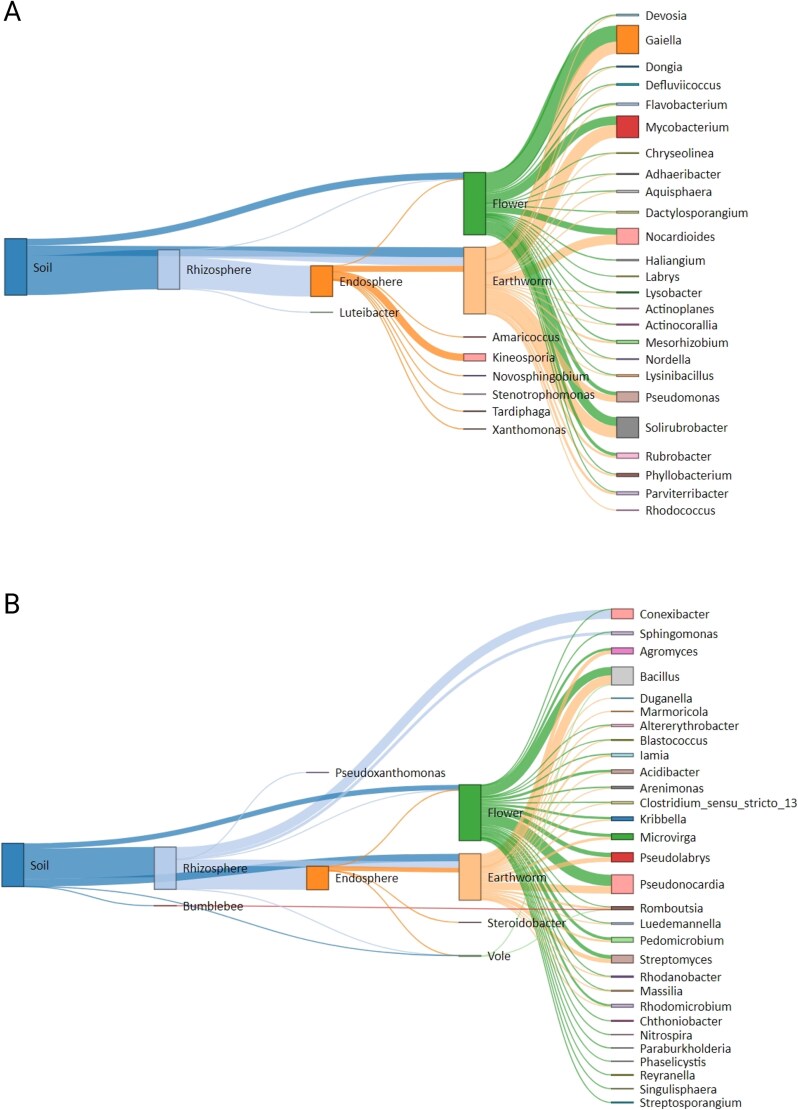
Proportions of bacterial genera overlapping between source and different target compartments along the trophic chain analyzed by SourceTracker2 analysis. (A) Results based on genera identified as resilient taxa, (B) results based on genera identified as both, resilient and responding taxa. In both analyses, the source compartments were soil, rhizosphere and endosphere, while key target compartments were endosphere, flowers, earthworms and voles.

To examine microbial communities overlapping across trophic compartments, we performed SourceTracker2 analysis on a refined dataset containing both *resilient* and *responder*-taxa (Fig. 4). Potential microbial sources—including soil, rhizosphere, endosphere and flowers- were defined based on their positions in the trophic chain. All analyzed interactions are summarized in Table S6.

Within the phytosphere (using soil, rhizosphere and endosphere as source), overlaps were observed between soil and rhizosphere (57 genera), rhizosphere and endosphere (47 genera), and soil and flowers (53 genera). In earthworms, substantial microbial overlap was detected with soil (53 genera), rhizosphere (42 genera), and endosphere (32 genera). In contrast, voles exhibited minimal overlap, sharing only two genera with soil, rhizosphere, and endosphere, while bumblebees overlapped in one genus with soil and did not have any overlapping genera with flowers (Fig. 4).

While taxa classified as *resilient* only showed few overlaps across compartments (Fig. 4A), most overlapping genera identified by SourceTracker2 corresponded to *fertilization-responsive-core* genera (i.e., both resilient and responder taxa, Fig. 4B). Prominent *resilient* genera were overlapping across multiple compartments (excluding voles and bumblebees) included *Gaiella, Mycobacterium, Solirubrobacter, Nocardioides, Pseudomonas, Kineosporia, Rubrobacter, Mesorhizobium, Parviterribacter,* and *Phyllobacterium* (Fig. 4A). Likewise, *Pseudonocardia, Bacillus, Conexibacter, Pseudolabrys, Streptomyces, Agromyces, Microvirga, Pedomicrobium, Kribbella,* and *Acidibacter* emerged as the most prevalent *fertilization-responsive-core* genera spanning multiple compartments (Fig. 4B). Notably, *Bacillus* and *Romboutsia* were present in all belowground compartments, including voles. Selective overlap of *Rhizobium* and *Paenarthrobacter* was observed between soil, rhizosphere, and endosphere.

To test the hypothesis that pig slurry may introduce allochthonous communities, we analyzed the microbial composition of pig slurry and its overlap with all trophic compartments. We noted *Clostridium sensu stricto* 1, *Terrisporobacter*, *Pseudomonas*, *Turicibacter*, *Lactobacillus*, and *Romboutsia* as the dominant genera in pig slurry (Fig. S5). These genera were detected across the trophic chain at relative abundances of 1–5%. Four genera viz. *Clostridium sensu stricto* 1, *Terrisporobacter, Turicibacter,* and *Romboutsia* were overlapping between pig slurry and other compartments (Fig. S6). Among them, *Turicibacter* was detected in all compartments including bumblebees, while *Terrisporobacter* and *Romboutsia* were found in flowers and all belowground compartments, excluding voles.

Despite overlaps in microbial genera across compartments and treatments, CAP revealed clear separation of microbial communities by trophic compartment (PERMANOVA: *P = .*001, df = 6, R^2^ = 0.389) and by fertilization regime across all compartments (PERMANOVA: *P = .*001, df = 3, R^2^ = 0.030) (Fig. S7). Pairwise PERMANOVA showed that each trophic compartment hosted a distinct microbial community, with all comparisons significantly different (Table S7). Likewise, fertilization regimes differed significantly from one another regardless of compartment (Table S8). These results indicate that microbial communities were consistently shaped by both compartment and fertilization regime.

## Discussion

Intensified land use can have significant effects on grassland biodiversity, spanning from microbes to fauna. We investigated whether manure-based fertilizers influence microbiomes across the grassland trophic chain. Our findings revealed that pig slurry, in particular, induced marked, host-dependent shifts. The study demonstrates that fertilization alters microbial diversity and composition across the trophic chain, with the most pronounced effects observed in belowground compartments. The strongest shifts were noted in soil, rhizosphere, and voles, suggesting heightened susceptibility in these compartments. While microbial composition remained largely host-dependent, we identified a core set of fertilization-responsive genera across compartments, highlighting the interdependence of trophic levels and underscoring the need for a comprehensive evaluation of fertilization impacts.

### Impact of fertilization on microbial diversity across trophic compartments

Microbiome analyses revealed contrasting effects of fertilization on microbial alpha diversity: it increased in soil and voles, but declined in the rhizosphere. These opposing trends likely reflect differences in ecological and physiological dynamics. Fertilization introduces both nutrients and allochthonous microbes. While the rhizosphere is shaped by host-driven selective pressures, soil is more permissive, allowing introduced microbes to establish more easily and potentially boosting diversity under intensive fertilization. Although soil and rhizosphere are closely linked, we observed a gradual but significant decline in microbial diversity in the rhizosphere, from minimally fertilized control to pig slurry-fertilized sites. This decline was associated with higher nutrient availability and a reduction in plant-beneficial bacteria involved in nutrient cycling under intensive land use [3, 65–67]. Plants actively shape their root microbiomes through rhizo-deposition, selectively recruiting or excluding microbes [68, 69]. Indeed, fertilized sites in our study exhibited elevated phosphorus and nitrate (NO₃) levels, with the highest concentrations found on pig slurry-treated sites. Consistent with these findings, reduced nitrification and lower *amoA* gene (ammonia monooxygenase) abundance were observed in nutrient-enriched soils, particularly those treated with pig slurry and biochar [70]. These results suggest that nutrient enrichment indirectly influences microbial community assembly in the rhizosphere. Expanding on this, we observed contrasting effects of fertilization on above- and belowground trophic compartments, with alpha diversity most significantly impacted belowground, especially under pig slurry application. Similarly, Le Provost et al. [1] found divergent responses between above- and belowground compartments across a land-use gradient, including fertilization, in their analysis of over 4000 taxa from grassland ecosystems.

### Fertilization-induced shifts in microbial composition and soil properties

Fertilization also altered microbial community composition, with assemblages across all compartments clustering according to the fertilization regime. However, the strength of this effect varied by trophic compartment. In soil, macronutrients and micronutrients together accounted for over 50% of the variation in microbial community structure, with calcium, phosphorus, and nitrate being the most influential factors. These results suggest that fertilization shapes soil microbiomes primarily through indirect physicochemical changes, rather than the direct introduction of non-native microbial taxa. Despite differences in soil nutrient composition and nitrogen inputs across fertilization regimes, plant biomass, flower number, and root biomass did not show significant variation. This indicates that changes in microbial composition were driven more by soil nutrient availability than by plant growth responses. These findings reinforce the role of fertilization in shaping soil and root microbiomes, reducing microbial complexity through altered soil properties and the introduction of livestock-associated microbes [4, 5, 17, 20]. Our results are consistent with those of Tyrrell et al. [71], who reported that swine, bovine, and poultry manure negatively impacted soil and phyllosphere microbiomes, leading to altered microbial communities and increased abundance of ARGs and mobile genetic elements (MGEs). These findings further underscore the grass phyllosphere as a reservoir for ARGs and MGEs, which can confer resistance to clinically relevant antimicrobials.

### Cascading effects of microbiota alterations across trophic chain

We observed microbial overlap across the trophic chain, with particularly high overlap between soil and rhizosphere microbiomes, as well as between soil and earthworm microbiomes. These findings suggest that fertilization-induced changes in soil and rhizosphere microbiomes may also influence the microbiomes of associated detritivores, herbivores, and ground-nesting pollinators. This aligns with growing evidence of microbial transmission from soil through roots to plant shoots, and ultimately to pollinators via flower-pollinator or soil-pollinator interactions [72–74]. Consequently, disturbances to microbiomes from fertilization may trigger cascading effects throughout the trophic chain.

While it is often assumed that allochthonous communities displace indigenous ones, thereby altering community composition [4, 5, 70], our findings suggest an indirect effect on native microbial communities, likely mediated by fertilization-driven changes in soil nutrient conditions. However, we detected four pig slurry-associated genera—*Clostridium sensu stricto 1*, *Terrisporobacter*, *Turicibacter*, and *Romboutsia*—in multiple trophic compartments. Although the relative abundance of these genera was low, their repeated detection across compartments suggests their ability to establish in diverse environmental niches. These findings raise significant health concerns. Previous studies have shown that repeated pig slurry application increases the diversity and abundance of ARGs and mobile genetic elements (MGEs), indicating potential long-term ecological impacts [75]. Pig slurry-associated taxa carrying ARGs pose a risk of horizontal gene transfer across both environmental and host-associated microbiomes. Their presence in pollinators and small mammals—organisms interacting with various ecosystem components—further highlights their potential role as reservoirs or vectors of microbial and genetic material.

In this study, we did not observe the previously reported microbial overlap between flowers and bumblebees [37]. This absence of overlap may be attributed to land use-driven reductions in floral resource abundance and diversity, which could lead insect pollinators to modify their foraging behavior [76]. However, *Turicibacter*—a pig slurry-associated microbe—was detected in bumblebees, which may be explained by their ground-nesting behavior and direct contact with fertilized soil, facilitating microbial overlap between belowground and aboveground compartments. Flowers, visited by multiple pollinators, may act as microbial reservoirs and transmission hubs, potentially influencing pollinator health through the spread of fertilizer-associated taxa [37, 72–74]. Similarly, belowground animals, such as the common vole, ingest soil and plant-associated microbes, affecting their microbiomes and potentially serving as reservoirs for antibiotic-resistant bacteria [8, 25, 36]. Fertilization-driven shifts in microbial communities, reduced complexity, and microbial influx can compromise host health. Moreover, affected hosts could serve as reservoirs for microbes carrying ARGs, further aiding their dissemination [6–8]. The potential for fertilizer-driven spread of ARGs and MGEs through trophic interactions in grasslands is a significant concern.

Microbial overlap along the trophic chain may suggest the potential for transmission across successive levels, highlighting the need to understand these dynamics to fully assess the impact of fertilization on grassland ecosystems, host organisms, and human health within a One Health framework [16]. Understanding microbial transmission dynamics between trophic compartments and fertilizers remains a major challenge, shaped by complex interactions involving environmental conditions, host physiology, trophic relationships, and microbial resilience. Definitive evidence would require controlled indoor studies using radioactively or fluorescently labeled microbes—an approach not feasible in field settings. Nonetheless, our 16S rRNA amplicon sequencing provides strong indications of potential transmission pathways influencing microbiomes across interconnected trophic compartments and lays the foundation for future controlled experiments to track marked microbes. Interpreting these transmissions requires considering host specificity and the effects of introduced or displaced microbial communities, emphasizing the need for further research.

In summary, fertilization—especially intensive pig slurry application—significantly alters microbiomes across grassland trophic compartments. The varied responses observed among compartments, along with partial cascading of microbial changes along the trophic chain, support the eco-holobiont framework [27], emphasizing the interconnectedness of microbial communities and their potential implications for both animal and human health.

## Supplementary Material

Jetter_Jani_supplementary_revised_ycaf162

## Data Availability

The datasets generated during and/or analysed during the current study conducted in cooperation with the Biodiversity Exploratories program (DFG Priority Program 1374) are publicly available on GitHub (https://github.com/jk00ANI/Fertilization-impacts-microbiomes-along-the-grassland-trophic-chain/tree/9ed11ecadad8fd6b491dbffe77abdcfd9ff1747e/nutrientData) and on NCBI (Bioproject number: PRJNA1188490) [77, 78].
