# Supplementary material for: Fertilization impacts microbiomes along the grassland trophic chain
Source: ISME Commun. 2025 Sep 16;5(1):ycaf162. doi: 10.1093/ismeco/ycaf162 (PMC12503161; doi:10.1093/ismeco/ycaf162)
Supplement: Jetter_Jani_supplementary_revised_ycaf162 [file jetter_jani_supplementary_revised_ycaf162.pdf]

**Supplemental Material**

# Fertilization impacts microbiomes along the grassland trophic chain

Karoline Jetter[1,2,4], Kunal Jani[2,4,6*], Kerstin Wilhelm[2], Ulrike Stehle[2], Rostand Chamedjeu[1,2], Christian U. Riedel[1,2,5], Lena Wilfert[2,5], Patrick Schäfer[3,5], Simone Sommer[2,5*]

[1] Microbial Biotechnology, Department of Biology, University of Ulm, Germany

[2] Institute of Evolutionary Ecology and Conservation Genomics, University of Ulm, Germany

[3] Institute of Phytopathology, iFZ Research Centre for Biosystems, Land Use and Nutrition, Justus Liebig University, Gießen, Germany

[4] These authors contributed equally

[5] Senior author

[6] Lead contact

[*] Corresponding author

## Supplementary Methods

### Sampling of focus specimens along the trophic chain

Soil, plant (red clover, *Trifolium pratense*), earthworm, bumblebee (*Bombus lapidarius*) and vole (*Microtus arvalis* and *Arvicola amphibius*) samples were collected in grassland ecosystems subjected to different fertilization regimes to investigate the effects of fertilization on microbiomes along the trophic chain (Figure S1B). All grassland sites except sites fertilized with pig slurry are part of the Biodiversity Exploratories framework (https://www.biodiversity-exploratories.de)[1]. The pig slurry fertilized sites were selected in cooperation with local farmers on the Swabian Alb (Figure S1B).

Soil, earthworms and plants were sampled together in an area of approximately 10cm x 10cm around a red clover stem. Soil was sampled by collecting the top layer (to a depth of 15 cm) from the 10x10x15 cm ditch and sifting through a 0.1 cm kitchen sieve to remove larger stones and roots. Samples of red clover *Trifolium pratense* were selected by visual inspection, choosing a healthy individual with at least one open flower at anthesis; large plants, usually characterized by large root systems, were excluded to facilitate root processing. Plant samples were collected by removing the plant with its roots from the ditch in order to retain the plant roots by carefully removing the bulk soil adhering to the roots. Plant height and number of flowers per plant were also recorded. Roots and flowers were separated from the aboveground biomass and all samples were kept on ice during transport to the laboratory for further processing. Root fresh weight was recorded and aboveground biomass was dried for 5-6 days at 60°C before measuring aboveground dry biomass. Due to the extremely dry climatic conditions during the summer of 2022, all earthworms were collected from the same 10x10x15cm ditch regardless of taxonomic affiliation. Earthworm feces were collected by placing the earthworms in a petri dish with a damp paper towel overnight and then releasing the earthworms. Bumblebees of the species *Bombus lapidarius* were captured at the field sites using a plastic container when visiting a flower inside or close to the site. Voles were captured using Sherman live traps with a bait containing a mixture of maltol solution, oat flakes and cereals, and an apple slice. The traps were modified according to Mori et al.[2] by attaching half a Tetra Pack carton with an entrance hole to the trap opening, which was then placed on a vole burrow. Traps were set in the late evening and checked early the next morning. If a vole was caught, its feces were collected in 400µl nucleic acid preservation (NAP) buffer[3] and the voles were released after recording their weight and sex. All samples were transported to the laboratory on ice for further processing and stored at -20°C until further analysis.

**Sample processing**

Plant samples were separated into root, flower and aboveground biomass samples. Root samples were separated into endosphere and rhizosphere samples (see Figure S1B). The rhizosphere was

washed from the roots by shaking the whole root in 25 ml sterile water, removing the roots and centrifuging the solution at 4000 × g for 15 min to obtain microbial cells. Endosphere samples were obtained by shaking the root in 30 ml of 0.1% (v/v) Triton X-100 in $H_2O$ followed by washing off the Triton solution with 30 ml of sterile water. Roots were then air dried on sterile filter paper and cut into smaller pieces for extraction. Flowers were sonicated in 20 ml sterile PBS-Tween solution (1x, 0.15%) for 10 minutes to remove epiphytic microbes. Flower parts were removed with sterile tweezers and the solution was centrifuged at 3000 rpm for 10 minutes to pellet the microbial cells. Both root and flower samples were stored at -80°C until DNA extraction. Aboveground wet biomass was assessed before plant material was dried at 50°C for at least 7 days to determine aboveground dry weight.

Bumblebees were euthanized by freezing at -20°C and frozen bees were dissected under sterile conditions to extract the gut. Gut samples were frozen at -80°C until extraction. Vole and earthworm feces were stored at -20°C.

**DNA extraction, library preparation and 16S rRNA gene amplicon sequencing**

To analyze the microbial composition, DNA of root, earthworm feces, vole feces and soil samples were extracted using the *ZymoBIOMICS DNA Miniprep* kit (Zymo Research Europe, Germany), whereas the *ZymoBIOMICS DNA Microprep* kit was used to extract DNA of flower and bumblebee gut samples. All samples were extracted following the manufacturer's instructions and subjected to an initial bead beating step using ceramic beads provided by the kit to mechanically lyse bacterial cells. To break open the roots for the endosphere samples five steel beads (2.4 mm, Bio-Budget Technologies, Germany) were additionally added to each sample. Bead beating was carried out using a SpeedMill PLUS (Analytik Jena, Germany) running two 3-minute cycles of bead-beating interrupted by a 3 -minute break.

To assess the bacterial composition, the hypervariable V4 region of the 16S rRNA gene was amplified in a two-step polymerase-chain reaction (PCR) using the primers 515 F (5'-GTGCCAGCMGCCGCGGTAA-

3′) and 806 R (5′-GGACTACHVGGGTWTCTAAT-3′)[4]. To avoid amplifying high amounts of chloroplast and mitochondrial DNA in plant (endosphere and flower) and bumblebee gut samples, 10µM peptide nucleic acid (PNA)-DNA clamps (mPNA and pPNA in plant samples and mPNAs in bumblebee samples) were added to the first PCR reaction[5]. The PCR regime for these samples included one more annealing step for 30 seconds at 78°C after denaturation within the 30 cycles. PCR success for all samples was confirmed by agarose gel electrophoresis. Appended adapters for forward (CS1-515F) and reverse (CS2-806R) primers were used in order to construct the library using Standard Bio Tools chemistry (Access Array System for Illumina Sequencing Systems, Standard BioTools, USA)[6–8]. The second PCR reaction was performed to attach individual barcodes and adapters used for illumina sequencing. To account for potential sequencing artifices arising from the DNA extraction, a positive control mock community (Zymo Research Europe, Germany), as well as extraction blanks and PCR-negative controls, were also included as a part of the in-house Illumina Miseq sequencing run at the Institute of Evolutionary Ecology and Conservation Genomics at Ulm University in Germany.

**Soil nutrient assessment by electro-ultrafiltration**

EUF separates nutrients into two fractions based on different water pressures and membrane types: the first fraction represents nutrients immediately available to plants, and the second contains nutrients that are deliverable over time. For our analysis, we combined both the immediately available and deliverable fractions to calculate the total amount of nutrients for each site. We used the information of the content of eight macronutrients (P, K, S, N, nitrate, Ca, Mg, B) and four micronutrients (Mn, Zn, Cu, Fe) as well as the sodium content in soil samples for subsequent analyses.

**Bioinformatic sequence processing**

Sequence processing was conducted on Qiime2 (version 2022.8) using the DADA2 pipeline which incorporates primer removal, denoising, chimera removal and merging of paired-end reads[9,10]. Given the high number of samples and the samples being sequenced on two different runs, the denoising steps were conducted for each run separately. Files of the representative sequences, metadata files

and the dada2 output table were merged before taxonomical assignment. For the first run forward reads were trimmed at 30 and 240 bp and reverse reads were trimmed at 30 and 215bp. For the second run, forward reads were trimmed at 30 and 240bp, reverse reads were trimmed at 30 and 220bp. For taxonomical assignment the SILVA 138-99 database[11] using the q2-feature classifier classify-sklearn (version 0.24.1)[12] in QIIME2 was used and run on the combined dataset of both sequencing runs. A phylogenetic tree was constructed after all ASVs were aligned with mafft[13] (via q2-alignment) using fasttree[14] (via q2-phylogeny). Using the software Dendroscope (version 3.8.4)[15], the unrooted phylogenetic tree was re-rooted using an uncultured archaeal sequence (accession number KT433146.1). The rooted phylogenetic tree and the biom table, both generated in Qiime2, together with the metadata file were imported into R using the phyloseq package[16] and a phyloseq object was created to conduct all statistical analysis using the R language[17] within the R Studio interface (version 4.3.2, Posit team (2023). The data processing of the phyloseq object in R precluded filtering of unassigned taxa as well as taxa assigned to chloroplast and mitochondria on family, class or order level and non-bacterial sequences. It was followed by filtering of samples by retaining the samples with non-zero reads.

## Supplementary Results

### Fertilization affects microbial composition of different trophic compartments

The fertilizer effects on microbial composition were more prominent in all trophic compartments at the genus level. Across belowground compartments, the most prominent differences in fertilized sites compared to control sites were lower abundances in *Bacillus* and higher abundances in *Pseudomonas* (Figure S8). In soil, the predominant genera at control sites were *Bacillus* and *Rokubacteriales* whereas *Gaiella* was the dominant genus in fertilized sites. Although *Rokubacteriales* were one of the dominating genera in biogas-fertilized and cow/horse manure-fertilized as well as control sites, they

were present only in lower abundances in pig slurry-fertilized sites. Despite differences in abundance, *Pseudomonas* was the predominant genus in the rhizosphere in all fertilization regimes and in the endosphere in pig slurry-fertilized sites. *Kineosporia,* which dominated the composition of the endosphere in control, biogas digestate and cow/horse manure-fertilized sites, was far less abundant under pig slurry fertilization. *Flavobacterium* and *Bacillus* dominated the earthworm composition and *Solirubrobacter* were more abundant in fertilized sites as compared to control sites. Microbial composition in voles was dominated by *Ruminococcus* and two *Clostridia* groups in all treatments. *Gastranaerophilales* were present only in fertilized sites while *Roseburia* and *Acetatifactor* were less abundant in fertilized sites. In flower compartments, *Massillia* and *Ralstonia* were dominant in samples collected on biogas and pig slurry-fertilized sites, while *Duganella,* which dominated samples from control sites, were less abundant in fertilized sites. In bumblebees, where *Gilliamella* were most abundant in all fertilization regimes, *Pseudomonas* were more abundant and *Lactobacillus* were less abundant on fertilized sites compared to control sites. Genera dominating pig slurry composition were detected in all the compartments across the trophic chain with relative abundances between 1% and 5%, so these analyses suggest an indirect effect of fertilization on the microbiome compositions of the different compartments.

**Table S1: Sample size distribution of the study.** Number of samples included in the microbiome analyses in relation to the seven different trophic compartments and fertilization regimes. Samples marked with an asterisk (*) represent pools of fecal samples of one species due to low volume of material collected at one sampling site. All other samples represent individual non-pooled samples. Numbers in brackets indicate sample sizes per plot.

| | Control | Biogas digestate | Cow/horse manure | Pig slurry | total |
|---|---|---|---|---|---|
| **Soil** | 24 (4/4/4/4/4/4) | 24 (4/4/4/4/4/4) | 20 (4/4/4/4/4) | 24 (4/4/4/4/4) | 92 |
| **Rhizosphere** (*Trifolium pratense*) | 24 (4/4/4/4/4/4) | 24 (4/4/4/4/4/4) | 20 (4/4/4/4/4) | 24 (4/4/4/4/4) | 92 |
| **Endosphere** (*Trifolium pratense*) | 24 (4/4/4/4/4/4) | 24 (4/4/4/4/4/4) | 20 (4/4/4/4/4) | 24 (4/4/4/4/4) | 92 |
| **Earthworm** | 6* (1/1/1/1/1/1) | 6* (1/1/1/1/1/1) | 4* (1/1/1/1) | 6* (1/1/1/1/1/1) | 22* |
| **Vole** (*Microtus arvalis, Arvicola amphibious)* | 5 (4/1) | 16 (3/1/7/1/1/3) | 25 (6/1/3/2/6/7) | 3 (2/1) | 49 |
| **Flower** (*Trifolium pratense*) | 24 (4/4/4/4/4/4) | 24 (4/4/4/4/4/4) | 20 (4/4/4/4/4) | 24 (4/4/4/4/4) | 92 |
| **Bumblebee** *(Bombus lapidarius)* | 18 (2/3/4/3/3/3) | 13 (1/4/3/3/2) | 16 (4/4/2/4/2) | 4 (4) | 51 |
| **Pig Slurry** | 0 | 0 | 0 | 4 (1/1/1/1) | 4 |
| **Total** | 125 | 131 | 125 | 109 | 494 |

**Table S2: Statistical results assessing the differences in alpha diversity (Shannon index) between the four different fertilization regimes within the seven different trophic compartments.** Indicated are p-values of ANOVAs and post-hoc Tukey's HSD pairwise comparisons. Significant values are marked in bold.

| Comparison | Soil | Rhizosphere | Endosphere | Vole | Earthworm | Flower | Bee |
|---|---|---|---|---|---|---|---|
| Overall | **0.029** | **0.001** | 0.145 | **0.001** | 0.107 | 0.124 | 0.473 |
| Control-pig | 0.075 | **0.001** | - | 0.091 | - | - | - |
| Control-biogas | 0.981 | 0.987 | - | **0.001** | - | - | - |
| Control-cow | 0.959 | 0.060 | - | **0.003** | - | - | - |
| Pig-biogas | **0.029** | **0.001** | - | 0.881 | - | - | - |
| Pig-cow | 0.262 | **0.049** | - | 0.999 | - | - | - |
| Biogas-cow | 0.826 | **0.026** | - | 0.549 | - | - | - |

**Table S3: Statistical results assessing differences in beta diversity (Bray-Curtis distances) between the four different fertilization regimes within the seven different trophic compartments.** P-values are given for PERMANOVA and pairwise PERMANOVA comparisons. Significant values are marked in bold.

| Comparison | All | Soil | Rhizo-sphere | Endo-sphere | Vole | Earth-worm | Flower | Bee |
|---|---|---|---|---|---|---|---|---|
| Overall | **0.001** | **0.001** | **0.001** | **0.001** | **0.001** | **0.005** | **0.001** | **0.001** |
| Control-pig | **0.001** | **0.001** | **0.001** | **0.001** | 0.656 | **0.021** | **0.001** | 0.229 |
| Control-biogas | **0.001** | **0.001** | **0.001** | **0.001** | **0.001** | 0.299 | **0.001** | **0.023** |
| Control-cow | **0.001** | **0.001** | **0.001** | **0.002** | **0.001** | **0.005** | **0.001** | 0.144 |
| Pig-biogas | **0.001** | **0.005** | **0.001** | **0.007** | **0.003** | 0.134 | **0.002** | **0.005** |
| Pig-cow | **0.001** | **0.005** | **0.001** | **0.001** | **0.005** | 0.121 | **0.002** | 0.37 |
| Biogas-cow | **0.003** | 0.153 | **0.009** | **0.01** | **0.002** | 0.052 | **0.015** | **0.001** |

**Table S4: Statistical results assessing differences in nitrogen input and plant parameters between the different fertilization regimes.** P-values are given for Kruskal-Wallis and posthoc pairwise Wilcoxon tests. Significant values are marked in bold.

| Comparison | Nitrogen input | Aboveground biomass | Root fresh weigh | Plant height | Number of flowers |
|---|---|---|---|---|---|
| Overall | **0.001** | 0.200 | **0.023** | **0.001** | 0.610 |
| Control-pig | **0.010** | 0.398 | 0.096 | **0.001** | 1.000 |
| Control-biogas | **0.006** | 0.301 | 1.000 | **0.001** | 1.000 |
| Control-cow | **0.013** | 1.000 | **0.046** | 1.000 | 1.000 |
| Pig-biogas | **0.017** | 1.000 | 0.330 | 1.000 | 1.000 |
| Pig-cow | **0.010** | 1.000 | 1.000 | **0.001** | 1.000 |
| Biogas-cow | **0.010** | 1.000 | 0.271 | **0.003** | 1.000 |

**Table S5: Resilient and responding identifiable genera and ASVs of the different compartments.** Genera have been filtered for unidentifiable taxa, that were not assigned until genus level or uncultured. Responding taxa are split for the paired comparison (all treatments compared to each other) and the contrasting comparison (all treatments against the control).

| | **Resilient** | **Responder** | |
|---|---|---|---|
| | | **Contrasting** | **Paired** |
| **Soil** | 47 (110 ASVs) | 9 genera (30 ASVs) | 3 genera (9 ASVs) |
| **Rhizosphere** | 62 (128 ASVs) | 6 genera (21 ASVs) | 1 genus (6 ASVs) |
| **Endosphere** | 99 (228 ASVs) | 17 genera (54 ASVs) | 3 genera (9 ASVs) |
| **Earthworm** | 67 (173 ASVs) | 3 genera (9 ASVs) | 1 Genus (3 ASVs) |
| **Vole** | 15 (138 ASVs) | 11 genera (51 ASVs) | 8 Genera (39 ASVs) |
| **Flower** | 12 (24 ASVs) | 5 genera (15 ASVs) | 0 |
| **Bumblebee** | 8 (19 ASVs) | 18 genera (57 ASVs) | 16 genera (48 ASVs) |

**Table S6: Set of interactions to decipher exchange of microbes across the trophic chain.** Compartments soil, rhizosphere or endosphere were considered as potential source while key target compartments were endosphere, flowers, earthworms and voles.

| | **Source** | | | |
|---|---|---|---|---|
| | Soil | Rhizosphere | Endosphere | Flower |
| **Sink** | Rhizosphere | Endosphere | Earthworm | Bumblebee |
| | Earthworm | Earthworm | Vole | |
| | Vole | Vole | | |
| | Flower | | | |

**Table S7: Statistical results assessing differences in beta diversity (Bray-Curtis distances) between the seven different trophic compartments across all fertilization regimes using the refined dataset containing only resilient and responding genera**. Indicated are p-values of Pairwise PERMANOVA comparisons. Significant values are marked in bold.

| | Soil | Rhizosphere | Endosphere | Earthworms | Voles | Flowers |
|---|---|---|---|---|---|---|
| Rhizosphere | **0.001** | | | | | |
| Endosphere | **0.001** | **0.001** | | | | |
| Earthworm | **0.001** | **0.001** | **0.001** | | | |
| Vole | **0.001** | **0.001** | **0.001** | **0.001** | | |
| Flower | **0.001** | **0.001** | **0.001** | **0.001** | **0.001** | |
| Bumblebee | **0.001** | **0.001** | **0.001** | **0.001** | **0.001** | **0.001** |

**Table S8: Statistical results assessing differences in beta diversity (Bray-Curtis) between the four different fertilization regimes across all trophic compartments using the refined dataset containing only resilient and responding genera.** Indicated are p-values of Pairwise PERMANOVA comparisons. Significant values are marked in bold.

| | Control | Biogas digestate | Cow/horse manure |
|---|---|---|---|
| Biogas digestate | **0.001** | | |
| Cow/horse manure | **0.001** | **0.007** | |
| Pig slurry | **0.001** | **0.001** | **0.001** |

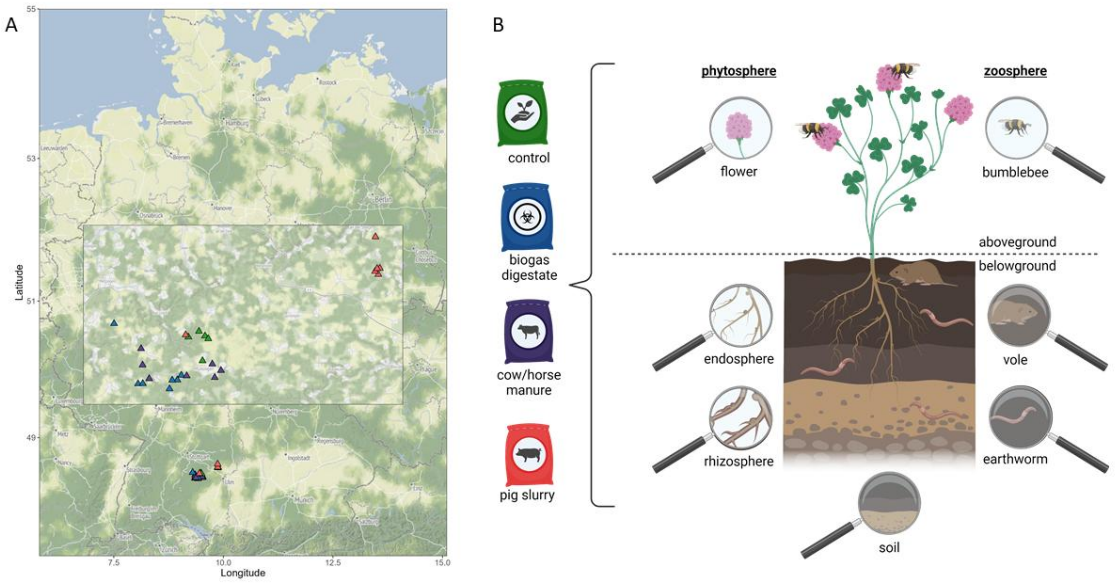


**Figure S1**: **Study design.** (A) Map of sampling sites located on the Swabian Alb in Baden-Württemberg, Germany, subjected to four different fertilization regimes (green: minimally fertilized control sites, blue: sites fertilized with biogas digestate, purple: sites fertilized with cow/horse manure, pink: sites fertilized with pig slurry). Map was created using R. (B) Study set up with seven different compartments of the trophic chain: soil, rhizosphere, root endosphere and flower (*Trifolium pratense)*, earthworm, vole (*Microtus arvalis, Arvicola amphibious*) and bumblebee (B*ombus lapidarius*) as well as the four different fertilization regimes: minimally fertilized control, biogas digestate fertilization, cow/horse manure fertilization and pig slurry fertilization. Compartments of the trophic chain can be grouped either in aboveground (flower and bumblebee) and belowground (soil, rhizosphere, endosphere, vole and earthworm) or into phytosphere compartments (rhizosphere, endosphere and flower) and zoosphere compartments (earthworm, vole, bumblebee).

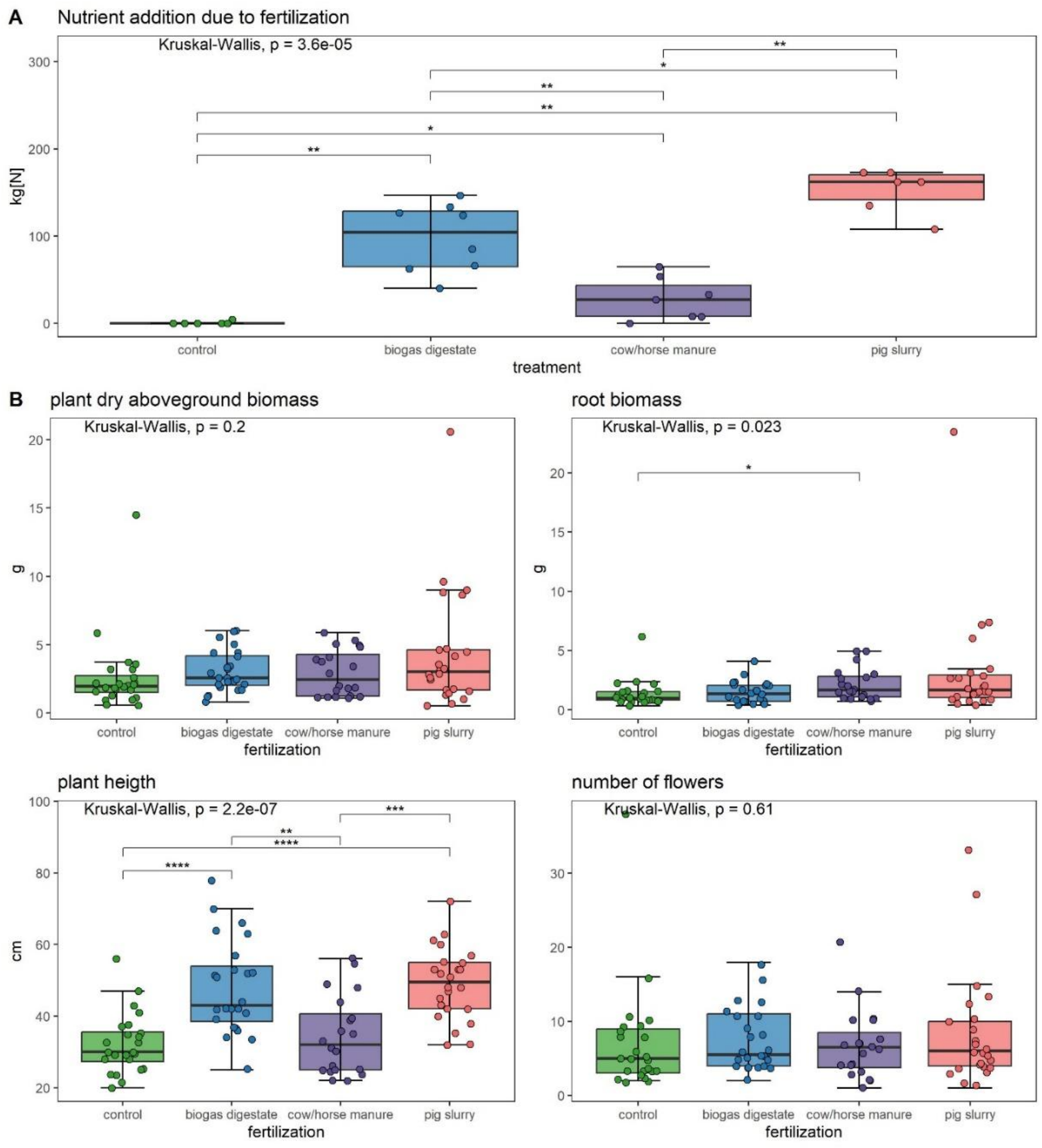


**Figure S2: Nitrogen addition (A) and plant growth parameters (B).** Amounts of nitrogen added to the respective field sites (A) was based on information from the land owners and the mean value of the sampling year and the last 4 years before sampling (2018-2022) was plotted. Organic fertilizers were converted into amount of nitrogen using standardized values from the LWK Nordrhein-Westfalen (2014) and the LWK Baden-Württemberg (2012), which are consistently used within the Biodiversity Exploratories' framework. Plant growth parameters aboveground dry biomass, root fresh biomass, plant height and number of flowers (B) were assessed for every plant collected from the field. In order to assess differences between the four fertilization regimes, Kruskal-Wallis tests followed by pairwise Wilcoxon post hoc tests were used.

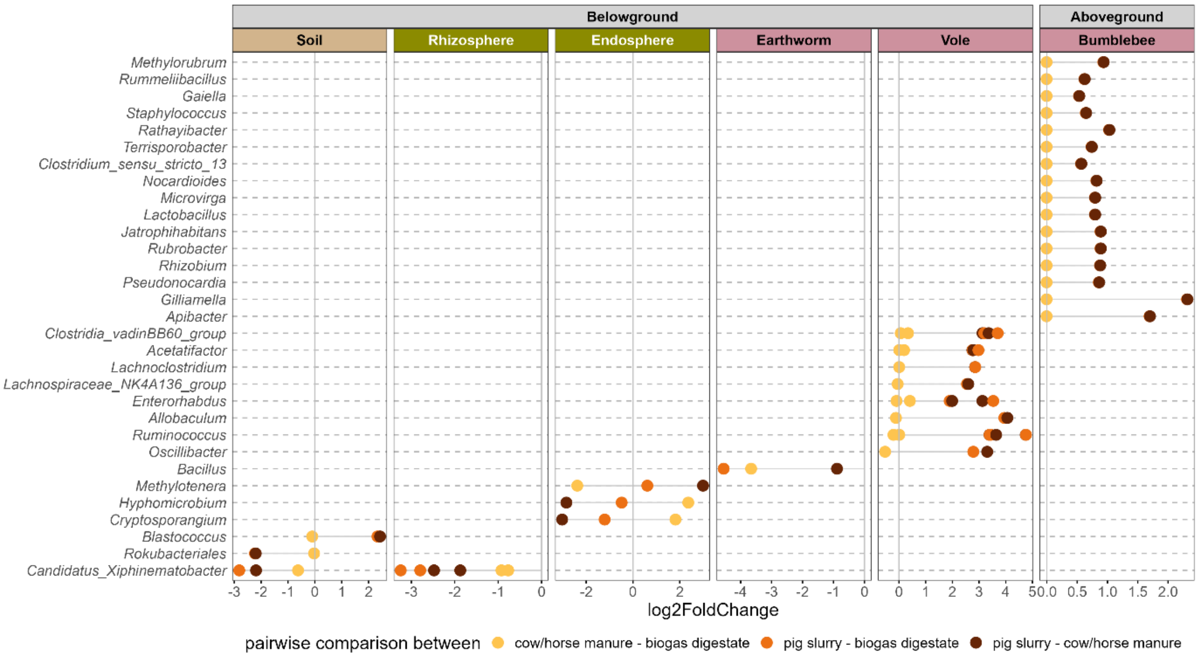


**Figure S3: Bacterial genera with abundance changes in response to fertilization in different trophic compartments.** Pairwise comparisons were made between cow and horse manure/biogas digestate (yellow), between sites fertilized with pig slurry/biogas digestate (orange) and between sites fertilized with pig slurry/cow and horse manure (brown) using ANCOMBC2. Flowers did not have any identifiable genera being significantly different for the tested comparisons and are hence not shown. Positive values of log2fold change represent genera that are more abundant in the second partner in the comparison; negative values represent genera that are more abundant in the first partner in the comparison.

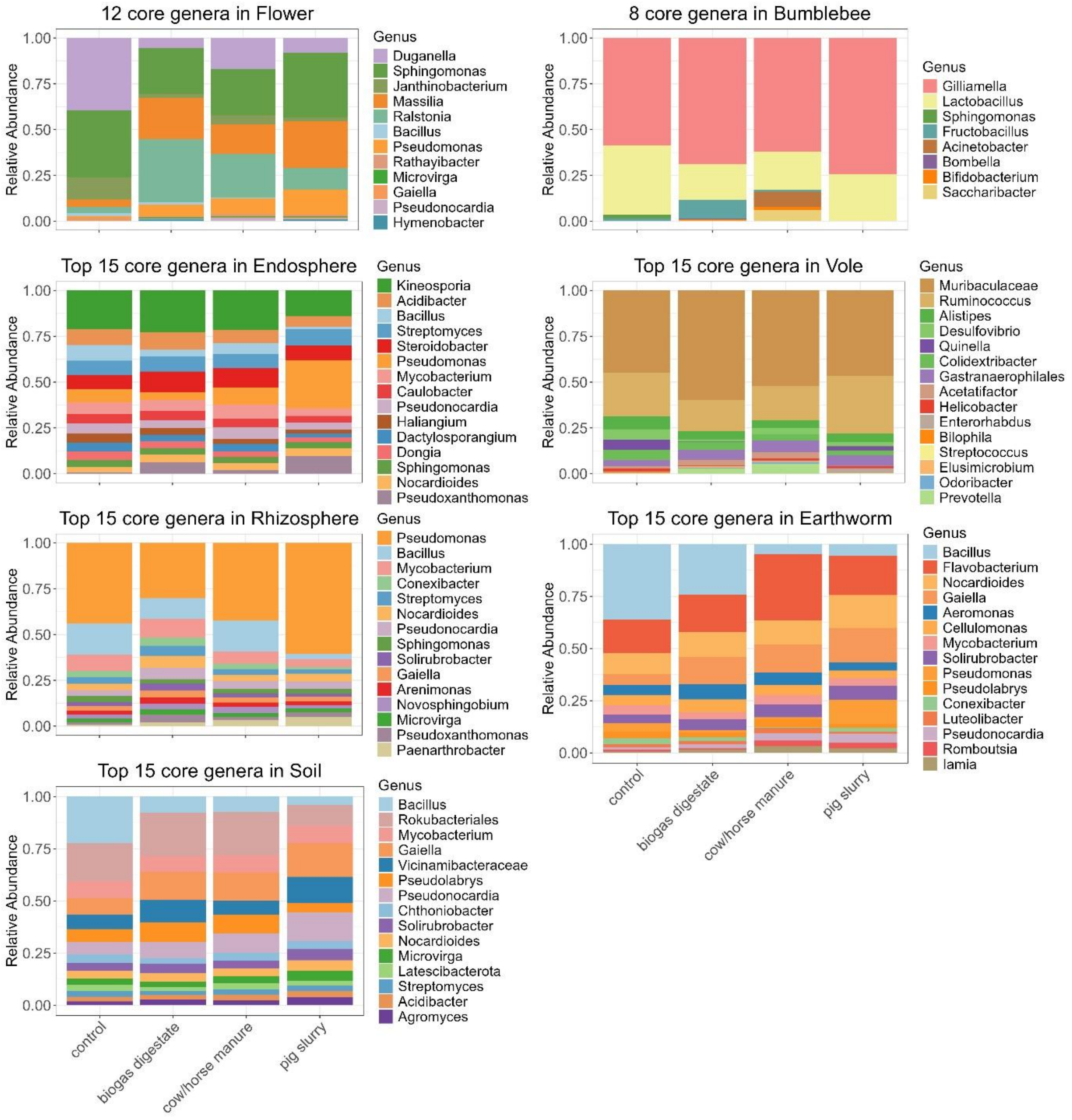


**Figure S4: Composition bar charts of the core genera in the different compartments along the trophic chain.** Indicated are the 15 most abundant core genera in soil, rhizosphere, endosphere, earthworms and voles, as well as the 12 core genera in flowers and 8 core genera in bumblebees in samples collected from sites subjected to the four different fertilization regimes (minimally fertilized control sites, biogas digestate fertilized sites, cow/horse manure fertilized sites and pig slurry fertilized sites).

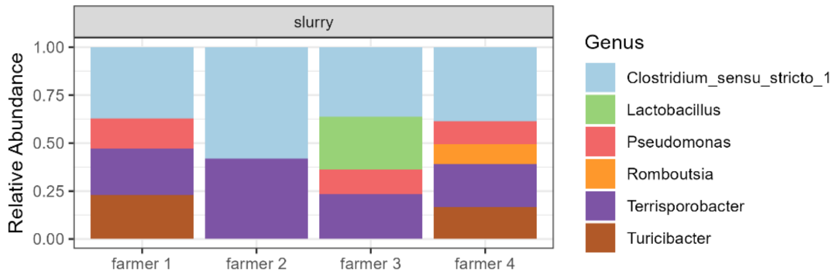


**Figure S5**: **Bacterial composition of the pig slurry.** Bacterial composition of pig slurry samples obtained from farm sites before application to the field indicating genera with a minimum abundance of 5%. Data represents individual samples.

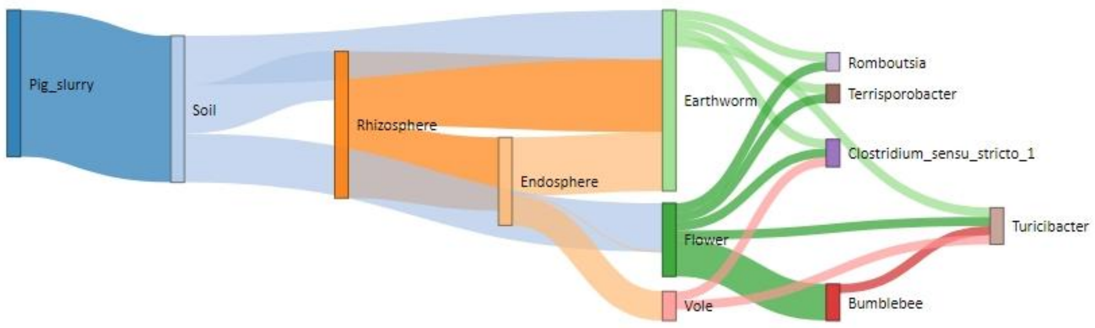


**Figure S6**: **Sourcetracker analysis showing the proportions of bacterial genera from pig slurry shared between compartments along the trophic chain.** Pig slurry was the primary source to all subsequent compartments, i.e. soil, rhizosphere, endosphere, while the main target compartments were earthworms, voles and flowers.

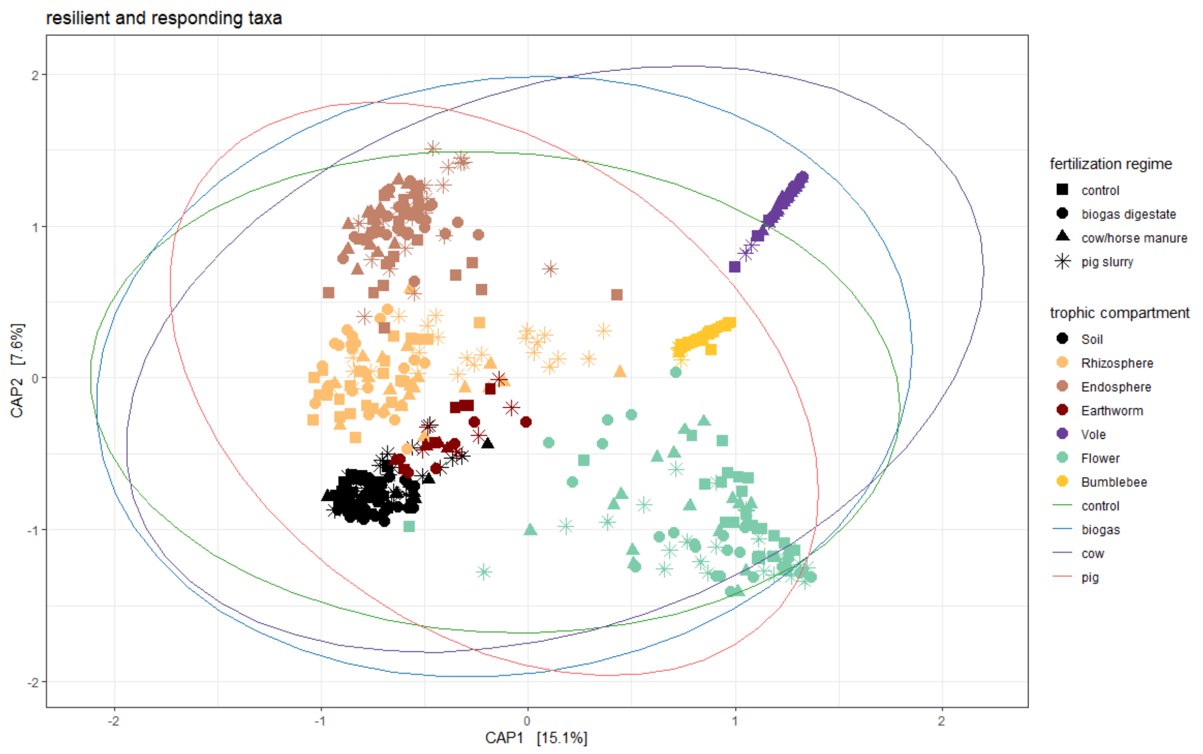


**Figure S7: Two-dimensional illustration of the refined dataset containing resilient and responder taxa identified by CAP analysis**. The different trophic compartments are indicated by colors and the fertilization regimes by different shapes.

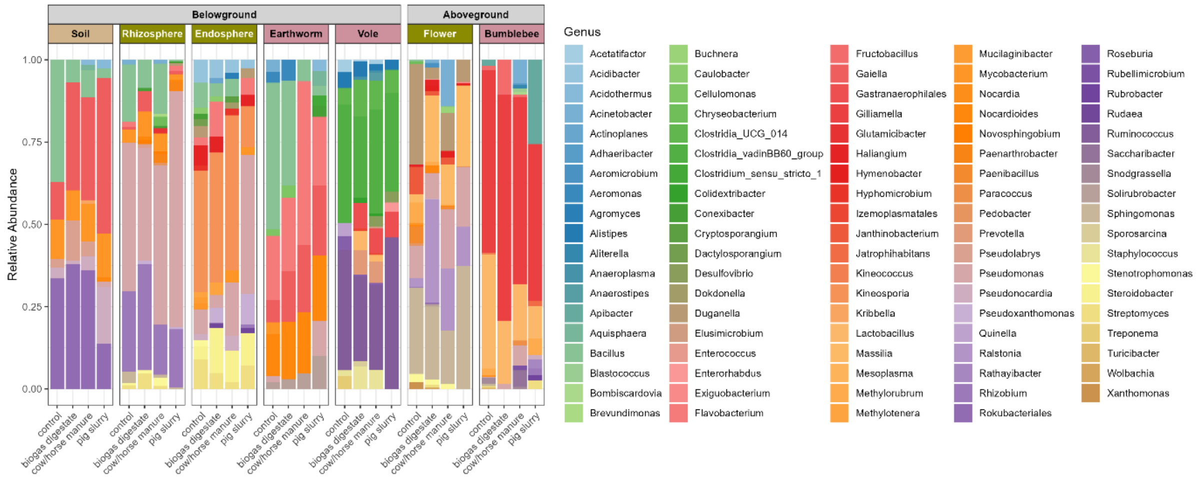


**Figure S8: Bacterial composition of the different compartments along the trophic chain.** Indicated are genera with a minimum abundance of 5%. Phytosphere compartments are marked in green, zoosphere compartments are marked in pink, soil is marked in brown. Data was pooled for plotting based on the fertilization regime within each compartment. Every fertilization regime contains up to 6 plots with 4 samples each, i.e. each bar represents a pool of up to 24 samples (see Table S1 for sample sizes).

**References**


1. Fischer, M., Bossdorf, O., Gockel, S., Hänsel, F., Hemp, A., Hessenmöller, D., Korte, G., Nieschulze, J., Pfeiffer, S., Prati, D., et al. (2010). Implementing large-scale and long-term functional biodiversity research: The Biodiversity Exploratories. Basic Appl Ecol *11*, 473–485. https://doi.org/10.1016/j.baae.2010.07.009.

2. Mori, E., Allegrini, C., and Bertolino, S. (2020). Modified live traps increase capture success of semifossorial voles in Alpine meadows. Mammalia *84*, 357–360. https://doi.org/10.1515/mammalia-2019-0098.

3. Camacho-Sanchez, M., Burraco, P., Gomez-Mestre, I., and Leonard, J.A. (2013). Preservation of RNA and DNA from mammal samples under field conditions. Mol Ecol Resour *13*, 663–673. https://doi.org/10.1111/1755-0998.12108.

4. Caporaso, J.G., Lauber, C.L., Walters, W.A., Berg-Lyons, D., Lozupone, C.A., Turnbaugh, P.J., Fierer, N., and Knight, R. (2011). Global patterns of 16S rRNA diversity at a depth of millions of sequences per sample. Proc Natl Acad Sci U S A *108*, 4516–4522. https://doi.org/10.1073/pnas.1000080107.

5. Víquez-R, L., Fleischer, R., Wilhelm, K., Tschapka, M., and Sommer, S. (2020). Jumping the green wall: The use of PNA-DNA clamps to enhance microbiome sampling depth in wildlife microbiome research. Ecol Evol *10*, 11779–11786. https://doi.org/10.1002/ece3.6814.

6. Fackelmann, G., Gillingham, M.A.F., Schmid, J., Heni, A.C., Wilhelm, K., Schwensow, N., and Sommer, S. (2021). Human encroachment into wildlife gut microbiomes. Commun Biol *4*, 800. https://doi.org/10.1038/s42003-021-02315-7.

7. Heni, A.C., Fackelmann, G., Eibner, G., Kreinert, S., Schmid, J., Schwensow, N.I., Wiegand, J., Wilhelm, K., and Sommer, S. (2023). Wildlife gut microbiomes of sympatric generalist species respond differently to anthropogenic landscape disturbances. Anim Microbiome *5*. https://doi.org/10.1186/s42523-023-00237-9.

8. Fleischer, R., Jones, C., Ledezma-Campos, P., Czirják, G., Sommer, S., Gillespie, T.R., and Vicente-Santos, A. (2024). Gut microbial shifts in vampire bats linked to immunity due to changed diet in human disturbed landscapes. Science of the Total Environment *907*. https://doi.org/10.1016/j.scitotenv.2023.167815.

9. Evan Bolyen, Jai Ram Rideout, Matthew R. Dillon, Nicholas A. Bokulich, Christian C. Abnet, Gabriel A. Al-Ghalith, Harriet Alexander, E.J.A., Manimozhiyan Arumugam, Francesco Asnicar, Yang Bai, et al. (2019). Reproducible, interactive, scalable and extensible microbiome data science using QIIME 2. Nat Biotechnol *37*, 852–857. https://doi.org/10.1038/s41587-019-0190-3.

10. Callahan, B.J., McMurdie, P.J., Rosen, M.J., Han, A.W., Johnson, A.J.A., and Holmes, S.P. (2016). DADA2: High-resolution sample inference from Illumina amplicon data. Nat Methods *13*, 581–583. https://doi.org/10.1038/nmeth.3869.

11. Quast, C., Pruesse, E., Yilmaz, P., Gerken, J., Schweer, T., Yarza, P., Peplies, J., and Glöckner, F.O. (2013). The SILVA ribosomal RNA gene database project: Improved data processing and web-based tools. Nucleic Acids Res *41*. https://doi.org/10.1093/nar/gks1219.

12. Bokulich, N.A., Dillon, M.R., Bolyen, E., Kaehler, B.D., Huttley, G.A., and Caporaso, J.G. (2018). q2-sample-classifier: machine-learning tools for microbiome classification and regression. J Open Res Softw *3*. https://doi.org/10.21105/JOSS.00934.

13. Katoh, K., Misawa, K., Kuma, K.-I., and Miyata, T. (2002). MAFFT: a novel method for rapid multiple sequence alignment based on fast Fourier transform. Nucleic Acids Res *30*, 3059–3066. https://doi.org/https://doi.org/10.1093/nar/gkf436.

14. Price, M.N., Dehal, P.S., and Arkin, A.P. (2010). FastTree 2 - Approximately maximum-likelihood trees for large alignments. PLoS One *5*. https://doi.org/10.1371/journal.pone.0009490.

15. Huson, D.H., Richter, D.C., Rausch, C., Dezulian, T., Franz, M., and Rupp, R. (2007). Dendroscope: An interactive viewer for large phylogenetic trees. BMC Bioinformatics *8*. https://doi.org/10.1186/1471-2105-8-460.

16. McMurdie, P.J., and Holmes, S. (2013). Phyloseq: An R Package for Reproducible Interactive Analysis and Graphics of Microbiome Census Data. PLoS One *8*. https://doi.org/10.1371/journal.pone.0061217.

17. R Core Team (2023). R: A Language and Environment for Statistical Computing. Preprint at R Foundation for Statistical Computing.
